# Biochemical Properties of Gastrokine-1 Purified from Chicken Gizzard Smooth Muscle

**DOI:** 10.1371/journal.pone.0003854

**Published:** 2008-12-05

**Authors:** Karim Hnia, Cécile Notarnicola, Pascal de Santa Barbara, Gérald Hugon, François Rivier, Dalila Laoudj-Chenivesse, Dominique Mornet

**Affiliations:** INSERM ERI 25 “Muscle et Pathologies”, Université Montpellier 1, EA 4202, CHU Arnaud de Villeneuve, Montpellier, France; University of Oldenburg, Germany

## Abstract

The potential role and function of gastrokine-1 (GNK1) in smooth muscle cells is investigated in this work by first establishing a preparative protocol to obtain this native protein from freshly dissected chicken gizzard. Some unexpected biochemical properties of gastrokine-1 were deduced by producing specific polyclonal antibody against the purified protein. We focused on the F-actin interaction with gastrokine-1 and the potential role and function in smooth muscle contractile properties.

**Background:**

GNK1 is thought to provide mucosal protection in the superficial gastric epithelium. However, the actual role of gastrokine-1 with regards to its known decreased expression in gastric cancer is still unknown. Recently, trefoil factors (TFF) were reported to have important roles in gastric epithelial regeneration and cell turnover, and could be involved in GNK1 interactions. The aim of this study was to evaluate the role and function of GNK1 in smooth muscle cells.

**Methodology/Principal Findings:**

From fresh chicken gizzard smooth muscle, an original purification procedure was used to purify a heat soluble 20 kDa protein that was sequenced and found to correspond to the gastrokine-1 protein sequence containing one BRICHOS domain and at least two or possibly three transmembrane regions. The purified protein was used to produce polyclonal antibody and highlighted the smooth muscle cell distribution and F-actin association of GNK1 through a few different methods.

**Conclusion/Significance:**

Altogether our data illustrate a broader distribution of gastrokine-1 in smooth muscle than only in the gastrointestinal epithelium, and the specific interaction with F-actin highlights and suggests a new role and function of GNK1 within smooth muscle cells. A potential role via TFF interaction in cell-cell adhesion and assembly of actin stress fibres is discussed.

## Introduction

Gastrokine-1 is a rather novel protein that is highly expressed in normal stomach and located in the superficial gastric epithelium. Gastrokine-1 is abundantly and specifically expressed in superficial gastric epithelium [1] but its function is not yet known. The current postulated role of gastrokine-1 in mucosal protection was deduced from its cellular localisation in the gastric epithelium. In previous reports, gastrokine-1 (GKN1), named before AMP-18 [2], [3] or CA11 [4], [5], was already known to be highly expressed in gastric antrum mucosa cells [6], [7], [8]. Previously generated mRNA expression profiles of gastric carcinoma were compared with those of normal gastric antrum and led to the identification of the gastrokine-1 transcript, which accounted for around 1% of the total mRNA in normal stomach but was absent from gastric cancers. Gastrokine-1 shows strong evolutionary conservation in the human, mouse, rat, cow, and pig. Sequence gene analysis (accession number: BK0017373) showed that the human transcript contains two potential translation start sites (ATG). The first would generate a 199 amino acid product lacking the signal peptide, unlike the second ATG, which is in-frame, 42 bp downstream, and has a more favourable kozak context for translation initiation. Comparison with other species revealed homology around the second site only, and its mature product is predicted at 18 kDa. Micro-sequencing of the native pig protein by Martin et al. [6] confirmed that the amino terminal residue was that predicted after signal peptide cleavage. The second ATG is therefore functional. The theory that gastrokine-1 is secreted is supported by some reported evidence of its localisation in cytoplasmic vacuoles. Furthermore, using immunohistochemistry and Western blot detection, Oein et al. [1] and Toback et al. [2] showed that gastrokine-1 was present in mucus at the gastric luminal surface.

Extensive tissue profiling by Northern blotting, in situ hybridisation and immunohistochemistry, showed that gastrokine-1 is highly and specifically expressed in native and metaplastic gastric epithelium, but such studies are restricted to gastro-intestinal tissues and a few other tissues such as liver, testis and placenta [1]. Gastrokine-1 is absent from gastric carcinomas [7] as well as from precursor lesions of intestinal metaplasia. Previous studies with a smaller range and number of samples revealed the specificity of this protein for gastric type epithelium and the absence from other normal epithelia. However, two studies using commercial sources of RNA or cDNA suggested that gastrokine-1 might also occur at low levels in normal placenta, uterus, liver, kidney, pancreas, and adrenal and salivary glands.

Protein sequence analysis predicted the signal peptide, with post-translational cleavage, 20 amino acid peptides from the N-terminus, with a conserved central domain structure named BRICHOS [8], which was postulated to contain two conservative cysteine residues that are possibly involved in intra- and/or inter-disulfide bridges. The putative association of gastrokine-1 with such a domain structure, and with at least three different possible functions, has been proposed [8] but has not been conclusively demonstrated.

To investigate the potential role of gastrokine-1 in smooth muscle cells, we present the first protocol for purification of native gastrokine-1 from chicken gizzard. We characterised the biochemical properties of gastrokine-1 with the aim of establishing some new unexpected biological functions. Using an original purification procedure, we identified a 20 kDa protein that is strongly expressed in chicken gizzard smooth muscle. Sequence blast showed that this protein corresponds to gastrokine-1 and the protein sequence showed that this protein contains one BRICHOS domain and at least two or possibly three transmembrane regions. IAEDANS labelling of gastrokine-1 showed that the protein contains a reactive cysteine residue. The full-length protein could be shortened into a protease resistant core by eliminating the first 12 N-terminal residues. Using a newly produced polyclonal antibody, we observed that gastrokine-1 is well distributed throughout the gastric epithelium, but also colocalizes with actin in smooth muscle cells. Moreover, by cross-linking and H-NMR approaches, we identified the actin binding site on the gastrokine-1 sequence involving the N-terminal acidic region of actin and demonstrated that the presence of tropomyosin favours gastrokine dimer formation and its association with actin filaments. Our results suggest that gastrokine-1 could regulate actin filaments inside the smooth muscle cytoplasm either to anchor the actin network to the epithelium cell membrane or to lock actin filaments for the actin-myosin interaction. Our results highlight a greater role of gastrokine-1 in cell-cell contact, in the actin network and in membrane anchorage in smooth muscles.

We also present, for the first time, a purification procedure to obtain gastrokine-1 as a 90% pure native protein from chicken gizzard muscle. A series of biochemical and structural studies on complete purified gastrokine-1 from chicken smooth muscle gizzard is also reported to investigate the properties of this protein and its possible relationship with other proteins present in smooth muscle. We demonstrate that chicken gastrokine-1 is a 20 kDa protein and contains an actin-binding site involving the N-terminal acidic region of actin and participates in actin assembly, as demonstrated by two methods, i.e. crosslinking and H-NMR techniques. Our results suggest that this gastrokine-1 could also be considered as a new actin binding protein and could have an effective locking role on actin filaments either by anchoring the actin network to the muscle cell membrane or by regulating actin-myosin interactions in smooth muscle cells.

## Materials and Methods

### Purification of gastrokine-1

The chicken gizzards used in this study were dissected from dead euthanized animals obtained from a slaughterhouse in Rémoulin (France). In details, fresh chicken gizzard (dissected and frozen at −80°C) was cut into small fragments (250 g) and mixed with 1 L of fresh extracting buffer (buffer A: KCl 300 mM, EDTA 1 mM, MgCl_2_ 0.5 mM and imidazole 50 mM, pH 6.9, freshly supplemented with soybean trypsin inhibitor 5 mg/L, phenyl methyl sulphate fluoride 5 mm and leupeptin 2 mg/L) in a Waring blendor 3 times for 5 min. The mixture was heated to 85°C for 4 min and cooled on ice before centrifugation at 4800 g for 30 min. The supernatant was collected and adjusted to 50% with ammonium sulphate under mild magnetic agitation. After centrifugation (4800 g for 30 min) the protein pellet was re-suspended (final volume about 25–30 ml) in buffer B (Tris HCl 20 mM, pH 7.5, EGTA 1,mM, DTT 0.5 mM). The homogenate was dialyzed against 1 L of buffer B overnight in cold room under mild magnetic agitation. After centrifugation (4800 g, 30 min) the dialyzed homogenate was equilibrated with buffer B and submitted to Trisacryl DEAE chromatography. The first eluted fractions were pooled, equilibrated with the buffer B and submitted to a Trisacryl CM chromatography. After a washing step with buffer B containing 10 mM NaCl, the retained proteins were specifically eluted with 100 mM NaCl. All 20 kDa enriched fractions were pooled and concentrated with Amicon cells using PM 10 membrane. Finally, the protein mixture was submitted to chromatography through a Sephacryl S200 column and factions containing only the 20 kDa protein were pooled and analysed by SDS-PAGE and Western blot. Protein concentrations were estimated by the BCA protein assay (Pierce).

Usual preparation, starting from 100 g of chicken gizzard, we used this method to purify about 10 mg of gastrokine-1 to about 90% purity.

### Reactive cysteine labelling of gastrokine-1

The concentration of pure gastrokine-1 and/or its trypsin proteolytic fragment was estimated using the BCA kit (Pierce). The thiol content of fresh unmodified gastrokine-1 was measured using 5-5′-dithiobis (2-nitrobenzoic acid) in the presence of 4 M guanidinium chloride. We determined that the total gastrokine-1 protein contained about 2.3 reactive thiol groups, in agreement with the determined amino acid composition. N-iodoacetyl-N′-(5-sulfo-1-naphtyl) ethylenediamine (IAEDANS) was incorporated into gastrokine-1 using 10 molar excess of dye for 30 min in the dark and with 10 mM Tris HCl, 2 mM MgCl_2_, pH 7.5. The reaction was stopped using 10 mM DTE. Excess label was removed with 60% ammonium sulphate. After centrifugation, the fluorescent pellet was re-suspended in appropriate buffer and purified using P10 Sephadex chromatography before use.

### Limited proteolysis of gastrokine-1

Native or IAEDANS labelled gastrokine-1 (1 mg/ml) was treated using 1 mg fresh trypsin stock solution in distilled water (dilution 1/200 = E/S) in 0.5 mM DTE and 10 mM Tris HCl buffer, pH 7.5. After a time-course treatment of gastrokine-1, fractions were analysed by SDS-PAGE with Coomassie blue dye for native protein or by SDS-PAGE under UV light.

### Cross-linking

Fresh protein solutions (1 mg/ml = gastrokine-1; mixture of gastrokine-1/F-actin = 1/7, or gastrokine-1/tropomyosin/F-actin = 1/1/7) were dialyzed in 25 mM MES buffer, pH 7.0, and cross-linking experiments were initiated by adding 10 mM EDC freshly dissolved in deionised water. The EDC-treated protein solutions were analyzed in SDS-PAGE 5–18% gradient gel. When suitable, F-actin was first treated with IAEDANS to incorporate fluorescent label on cysteine 374 according to previously described conditions [9]. The gel is first viewed under UV light and then stained by Coomassie blue dye to compare the gel patterns.

### H-NMR

Proton-NMR spectra were recorded at 500 MHz on a Bruker AMX instrument. The proton resonances of the actin peptide (5 µl final volume containing 1 µl of each stock solution peptide at 1 mg/ml) were assigned using standard 2-D NMR procedures for spin system identification (COSY, 1H-1H RELAY and TOCSY) and sequential consecutive determination (NOESY and ROESY). Experiments were typically acquired using 2 K points and 512 and 256 blocks (RELAY/TOCSY or NOESY/ROESY, respectively). The majority of these studies were carried out at pH 6.9 at an ambient probe temperature of 285 K. Binding titrations involved the addition of small aliquots of gastrokine-1 stock solution (5 mg/ml) to reach concentrations corresponding to a 1/20 final molar ratio. Interactions were routinely detected by using difference spectroscopy to highlight broadening of the enhanced line upon the addition of gastrokine-1. The two-pulse spin echo method (r = 60 ms) was also used to better distinguish resonances in the one-dimensional spectrum.

### Antibodies

Polyclonal antibodies directed against gastrokine-1 or caldesmon, (using fresh and purified native proteins as antigen), were produced in the laboratory according to previously described protocols [10]. Smooth muscle actin was detected using the α-SMA commercial antibody (Santacruz Biotechnologies). Gastrokine-1 and caldesmon polyclonal antibodies were characterized as specifically detecting the protein bands only with Mr 20 kDa or 120 kDa, respectively.

### Immunohistochemistry and immunofluorescence

Timed fertilized white Leghorn eggs (Haas, France) were incubated at 38°C in a humidified incubator (Coudelou, France) until testing. Embryos were staged by embryonic day (E). Whole embryos or dissected gut tissues were fixed in 4% paraformaldehyde for 4 (at room temperature) to 18 (at 4°C) h before analysis. Fixed chick tissues were embedded in paraffin and blocks were cut into 3 to 5 µm sections and placed on Superfrost Plus slides (Fisher). Immunohistochemistry were performed using standard techniques. Briefly, endogenous peroxidase was blocked in 1.5% hydrogen peroxide for 30–60 min and all controls were obtained as previously described [11]. Primary antibodies were applied at the following dilutions: gastrokine-1 polyclonal antibody (1∶200); α-SMA monoclonal antibody actin (1∶100) and caldesmon polyclonal antibody (1∶50) and revealed by incubation for 1 h in ABC reagent according to the manufacturer's instructions (Dako) as previously described [11]. Unfixed cryostat sections (3–5 µm) of various embryonic chicken structures were incubated with specific primary antibodies for 1 h at room temperature. After washing with phosphate-buffer saline solution, sections were incubated with the secondary antibody (Cy3-conjugated goat anti-rabbit IgG, or with fluorescein-congugated goat anti-mouse antibody, Chemicon International, 1/4000). All sections were mounted with Mowiol and observed under a Nikon optiphot-2 microscope.

### SDS-PAGE and Western blotting

Purified proteins were generally adjusted to 1–2 mg/ml and a 50 µl aliquot was mixed with 50 µl of SDS buffer containing 0.01% bromophenol blue, 10% glycerol and 5% beta-mercaptoethanol. Samples were denatured for 5 min at 95°C. All protein samples were submitted in duplicate to SDS-PAGE. One resulting gel was Coomassie blue stained and the other was transferred onto 0.2 µm nitrocellulose membrane. Each blot was blocked in Tris-buffered saline with 0.1% Tween 20 (TBST) containing 3% bovine serum albumin (w/v). The corresponding membranes were incubated with gastrokine-1 primary antibody (1/1000 dilution) for 1 h at room temperature. After labelling, the membranes were washed in TBST and then incubated with a phosphatase-labeled second antibody (Jackson Immuno Research Laboratory, appropriate dilution). Antibody-bound proteins were detected with NBT/BCIP substrate.

## Results

### Purification and characterisation of gastrokine-1 from gizzard smooth muscle

**Figure 1:**
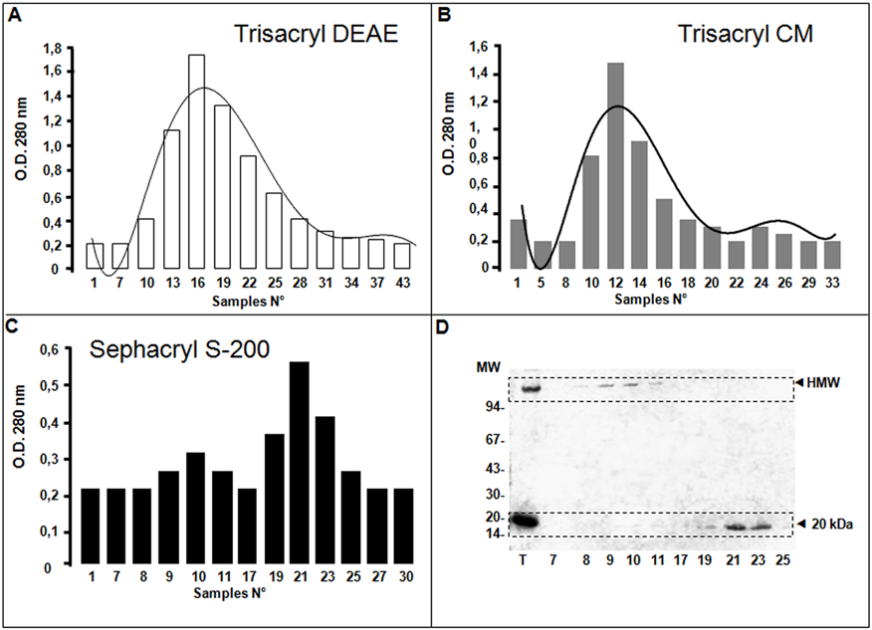
Purification of the 20 kDa protein contained in a conventional smooth muscle caldesmon preparation. Part A: the elution pattern under white histogram representation corresponds to Trisacryl DEAE chromatography and the elution pattern is shown and corresponds to the washing step of this chromatography (with buffer B). The eluted fractions from tubes 10 to 28 were pooled and then submitted to Trisacryl CM chromatography equilibrated in buffer B. After a washing step with buffer B containing 10 mM NaCl, the retained proteins were specifically eluted as shown in part B. Part B: the elution pattern shown in the grey histogram representation corresponds to Trisacryl CM treated with 100 mM NaCl and the 20 kDa protein enriched fraction (tube numbers 9 to 17) were pooled and concentrated with Amicon cells using a PM 10 membrane. The resulting sample of this concentrated fraction is shown in the first lane of the part D gel. Part C: the enriched 20 kDa preparation was submitted to Sephacryl S-200 chromatography and the elution profile recorded at OD 280 mm indicates, under the black histogram representation, that two proteins were separated into the corresponding fractions referred to as tubes 9 to 11 and tubes 19 to 25, respectively. Part D: corresponding to the PAGE stained by Coomassie blue of each eluted fraction, identified as numbered samples of this chromatography, providing access to separate proteins present in the initial enriched 20 kDa solution according to their respective molecular weights. Some 50 microliters samples of each fraction were denatured for 5 min at 100°C and then analyzed by electrophoresis in 0.1% SDS polyacrylamide slab gels using a 5–18% acrylamide gradient with the Hoefer commercial apparatus. The running buffer was 25 mM Tris HCl/glycine 192 mM in the presence of 0.1% SDS. The result of the Coomassie blue stained acrylamide gel is presented (part D) and showed a clear separation between fractions (HMW) contaminating high molecular weight proteins (tubes 9 to 11) and enriched 20 kDa protein (tubes 19 to 25). These fractions were pooled and concentrated with a PM 10 Amicon cell.

Most data related to gastrokine-1 is derived from studies on the gastric epithelium and more recently from the purification of gastric epithelial cell populations free of contaminating stromal elements. This was accurately achieved using laser capture microdissection, as described in a recent study [12]. In the present work, we intended to broaden the research field by using whole chicken gizzard smooth muscle to purify gastrokine-1. We reviewed the scientific background on biochemical protein preparation related to smooth muscle and found protocols addressed to the major expressed proteins contained in this tissue, such as myosin [13], [14], caldesmon [13], [14], [15], calmodulin [16], and smooth muscle myosin light chain kinase [17]. In our experiments, we have initiated purification steps to obtain pure caldesmon. During the first step of caldesmon purification, we detected the presence of an abundant protein, with an Mr of around 20 kDa, in the first eluted fractions during the Trisacryl DEAE chromatography washing step, whereas these eluted fractions are generally not used for caldesmon purification [18]. As shown in “Figure 1” (part A), the first eluted fractions were collected before the salt elution of caldesmon retained on the Trisacryl column was started. Briefly, the smooth muscle total protein extract is submitted to heat denaturation and then a crude protein pellet is obtained after ammonium sulphate precipitation and centrifugation steps (see Material and Methods section). The enriched-caldesmon preparation obtained was then submitted to anionic chromatography. At this step, according to the regular caldesmon purification protocol, the optical density at 280 nm must be recorded and return to baseline before performing a salt gradient to selectively obtain pure caldesmon. After recording the yellow-stained elution, we analyzed each fraction and pooled them as presented in “Figure 1” part A, and we obtained an enriched 20 kDa protein solution. Two selective chromatographs were used, as shown in “Figure 1” part B and C, and we selected the fractions containing the 20 kDa protein. The Trisacryl CM column allowed us to purify our 20 kDa protein preparation and, after concentration with an Amicon cell using PM10 membrane, the last chromatograph on Sephadex S-200 was analyzed by PAGE as presented in “Figure 1” part D. A final step of concentration allows us to obtain a purified 20 kDa protein preparation.

**Figure 2:**
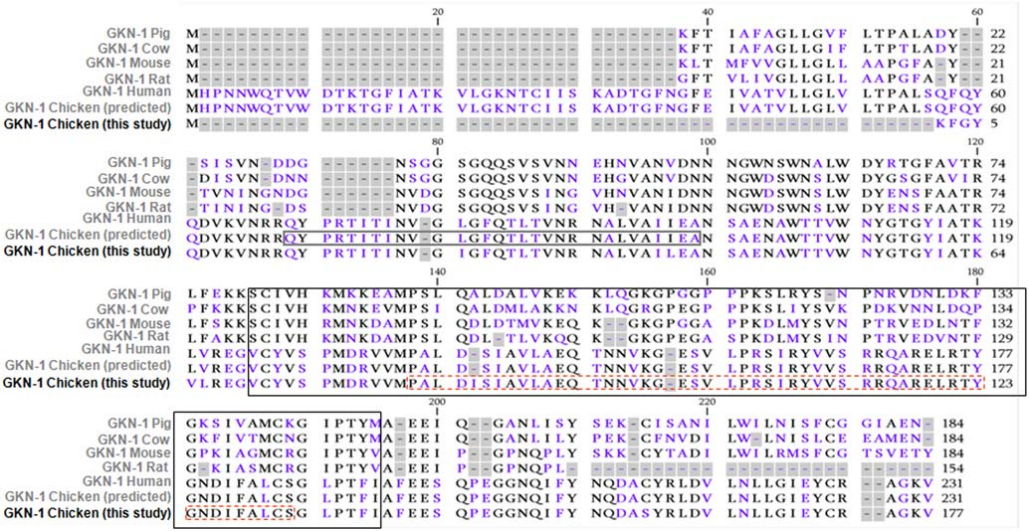
Sequence alignment for various gastrokine-1 proteins. In this representation, we have summarized the gastrokine-1 sequences already published in a previous work [1] and corresponding to gastrokine-1(GKN1) from pig (line 1), cow (line 2), mouse (line 3), rat (line 4), human (line 5) and Gallus gallus NCBI (line 6) as compared to our sequence obtained in this work (line 7). The sequence presented in this work was obtained by sequencing of isolated chicken gizzard GNK1 protein and the first CNBR peptide is underlined in a dashed box. It closely neighboured a methionine residue and was confirmed by a tryptic overlapping peptide with a sequence corresponding to VVMPALDISAVLAEQT. The N-terminal tryptic fragment of GNK1 (18 kDa protein) is underlined in a dark box. Note that this peptide mapping allowed us to obtain almost the entire gastrokine-1 sequence with only 12 residues missing, but after such alignment there was 65–61% homology between our 20 kDa isolated protein and gastrokine-1 protein from human, pig or mouse, and about 90% homology with the recently published Gallus gallus sequence. The BRICHOS domain is shown in the large black box.

The total amino acid composition of the purified 20 kDa protein was recorded and, as summarized in “Table 1”, this protein contained about 175 residues without any His, which is not frequently observed. Since there were only 2 Met residues and a few basic residues (4 Lys and 15 Arg), we submitted the 20 kDa protein to selective CNBR in a first step and to limited trypsin cleavage in a second step (as shown later in “Figure 2” and “Supplement S1” figure. After HPLC purification in conditions similar to those described in the “Table 1” legend, the obtained peptides were subjected to amino acid composition and sequence determinations. 200 to 300 picomoles of peptide were analyzed and only sequences with repetitive yields greater than 95% per cycle were considered as precluding unambiguous determination. A particularly long peptide was sequenced after CNBR cleavage up to step 50. The primary sequence of this peptide was:

**Table 1:** Amino acid composition of the purified 20 kDa protein.

Amino acid residues	Total 20 kDa protein	CNBR peptide
ASP	20	2
THR	11	2
SER	7	4
GLU	21	3
PRO	?	2
GLY	13	2
ALA	15	5
CYS	2	1
VAL	18	5
MET	2	0
ILE	11	3
LEU	15	5
TYR	10	2
PHE	6	1
HIS	0	0
LYS	4	1
ARG	14	6
TRP	ND	ND
GLN	ND	2
ASN	ND	3
Total	175	48

The pure 20 kDa protein obtained after concentration with an Amicon cell and using PM10 membrane was then purified by reversed-phase HPLC a using gradient liquid chromatography and a 4.6×75 mm Ultrapore RPSC C8 column. A linear gradient elution was performed in 60 min at a flow rate of 1 ml/min using solvent A: 0.08% trifluoroacetic acid in water, and solvent B: 0.08% trifluoroacetic acid in 60% acetonitrile. Peptide detections were monitored at 220 mm. Such purified 20 kDa preparation was either used as antigen to be injected in rabbit to obtain specific polyclonal antibody directed against the 20 kDa protein, or for amino acid composition determination. Amino acid analyses were performed on protein samples hydrolysed at 110°C under vacuum in 6 M HCl for 24 h using a Beckman Model 332B autoanalyzer. ND: not determined.

*PALDISIAVLAEQTNNVKGESVLPRSIRVVSRRQRAELRTYGNDIFALC.*

When submitted to protein sequence alignment, this peptide showed only one significant alignment with gastrokine-1 (GKN1). Amino acid sequencing of other peptides derived from limited trypsin cleavages and purified by HPLC were included and showed a significant alignment with the recently deduced gastrokine-1 sequence (“Figure 2”). It is of interest to note that our CNBR peptide described above was closely neighbouring a Met residue and corresponded to part of the BRICHOS domain described in each gastrokine-1 sequence. One conserved cysteine within the pair was not sequenced, possibly because its neighbouring region is rich in basic residues, i.e. the target of trypsin attack. However, the complete sequence of the BRICHOS domain was obtained in our peptide mapping (12 residues missing for a total of about 100 residues), with about 35% homology. The results presented above led us to assume that our purified 20 kDa protein was clearly the chicken gastrokine-1 protein. This protein was soluble in buffer containing KCl 300 mM, EDTA 1 mM, MgCl_2_ 0.5 mM and imidazole 50 mM, pH 6.9, and was found to be a heat stable protein (85°C for 4 min) that easily refolded. Our experiment also revealed that gastrokine-1 specifically precipitated ammonium sulphate by 60% and could be easily re-suspended as soluble material which led us to investigate some biochemical and biological properties of gastrokine-1.

### Biochemical properties of gastrokine-1

**Figure 3:**
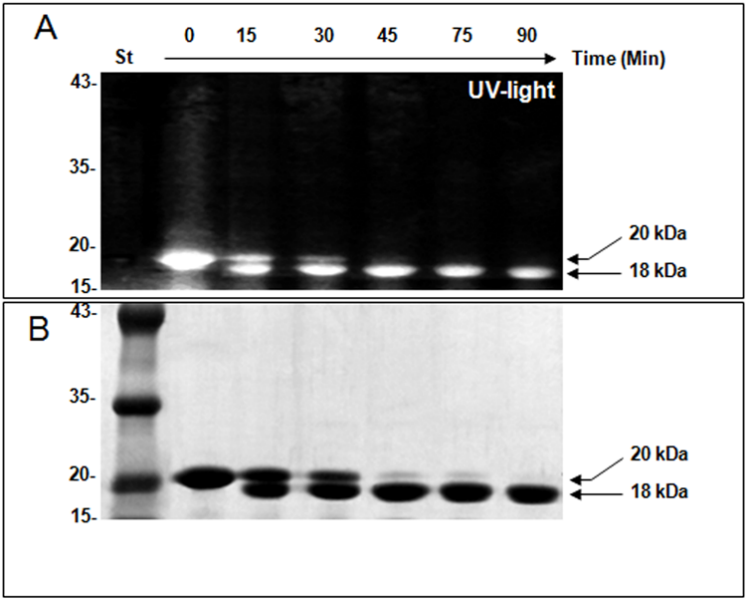
Fluorescent labelling of chicken gastrokine-1 and time course of limited trypsin cleavage. The digestion times are indicated in the top of the figure and corresponded to the trypsin time course of the purified 20 kDa preparation. Part A: the polyacrylamide slab gel was viewed under UV light. The 20 kDa fluorescent protein band was progressively converted and accumulated into a still fluorescent 18 kDa protein band. Part B: the same gel was viewed after Coomassie blue staining. The purified 20 kDa protein band was progressively converted into a stable 18 kDa protein band and remained stable after 90 min of trypsin attack. Then trypsin cleavage was stopped by adding a 2 molar excess of soybean trypsin inhibitor (STI) and the stable 18 kDa cleaved protein could be used in other experiments.

As shown by the amino acid sequence, gastrokine-1 contains four cysteine residues which could be important for the BRICHOS domain function [8]. We investigated the cysteine reactivity versus specific fluorescent labelling using IAEDANS (“Figure 3”). In our experimental conditions, the dye was covalently incorporated into the chicken gastrokine-1 and fluorescent GNK1 could be prepared and purified without excess of fluorescent dye, as detailed in the Materials and Methods. When viewed under UV light, purified GNK1 was detected as a fluorescent protein band with Mr 20 kDa, as expected, and even after tryptic cleavage the fluorescent cysteine residue remained associated with a stable 18 kDa cleaved fragment (“Figure 3” top part). In fact, time-course trypsin attack of native chicken gastrokine-1 (20 kDa) showed progressive conversion into the stable 18 kDa fragment (“Figure 3” bottom part). This clearly suggests that gastrokine-1 is a protease resistant protein and that trypsin treatment cleaves only the 12 N-terminal residues, as revealed by the amino acid sequencing data already presented in “Figure 2”. This is in agreement with the fact that some proteins containing the BRICHOS domain were cleaved in their N-terminal part [8]. On the other hand, our findings, in accordance with sequence analysis results, showed that gastrokine-1 contained cysteine residues and that one of them could be considered as a very reactive cysteine easily labelled by IAEDANS. When screening for the proteolytic product of gastrokine-1, this 18 kDa fragment was purified and sequenced (“Figure 2”: bold and italic sequence). According to the sequence homology, our results supported previous findings [19], indicating the presence of 3 transmembrane helices spanning residues 18–40, 59–79 and 110–127, as predicted with TMHMM [20]. However, the expected 14-amino acid mitogenic domain described previously [2] (“Figure 2” residues in italics, 100–113 in human) was only found to be about 30% conserved in the gastrokine-1 chicken sequence. The time course of tryptic cleavage was not dependent on the fluorescent modification because native purified gastrokine-1 gave a similar trypsin induced degradation profile, with a final 18 kDa resistant protein band (this is completely supported by “Supplement S1” figure, corresponding to the Coomassie blue stained gel for the time course of the native purified gastrokine-1 preparation).

### Gastrokine-1 co-localizes with F-actin

**Figure 4:**
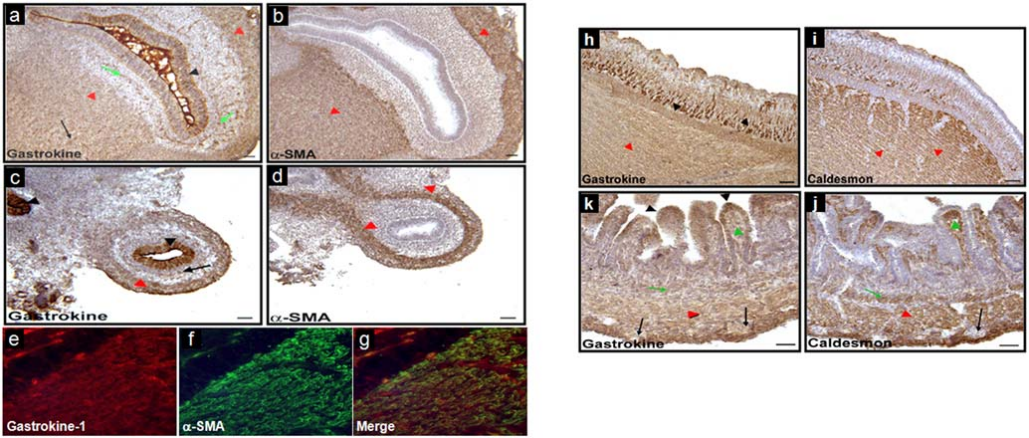
Gastrokine-1 expression during gastrointestinal development. Immunostaining for gastrokine-1 in E8 chick gizzard (a–b) and duodenum (c–d). Gastrokine was present in gastrointestinal epithelia (black arrowheads) and smooth muscle cells (red arrowheads). In addition, gastrokine was detected in isolated islets present in both smooth muscle (red arrows) and submucosal layers (green arrows). Immunostaining for alpha-SMA in E8 chick gizzard (b) and duodenum (d). Alpha-SMA was only present in smooth muscle cells (red arrowheads). Immunofluorescence for gastrokine (e) and alpha-SMA (f) in E8 chick duodenum. Gastrokine and alphha-SMA were present in the smooth muscle layers as shown in the merged image (g). In addition, gastrokine was present in duodenal epithelial cells (e, g) as observed in (b). Immunostaining for gastrokine in E16 chick gizzard (h) and duodenum (i). Gastrokine was present in gastrointestinal epithelia (black arrowheads) and smooth muscle cells (red arrowheads). Gastrokine was widely expressed in smooth muscle layers (h, i), but was not expressed in enteric nerve cells (black arrow), as observed in (i). Gastrokine expression was also observed in myofibroblasts in the lamina propria (green arrowhead) and in isolated islets (green arrow) present in the submucosa layer (i). Note that gastrokine was detected in basal epithelial cells in the gizzard (h) and in apical epithelial cells in the duodenum (i). Immunostaining for caldesmon in E16 chick gizzard (j) and duodenum (k). j, k, Caldesmon was present in gastrointestinal smooth muscle cells (red arrowheads). Caldesmon was widely expressed in the smooth muscle layers (j, k), but not expressed in enteric nerve cells (black arrow), as observed in (k). Caldesmon expression was also observed in myofibroblasts in the lamina propria (green arrowhead) and in the muscularis mucosa (red arrow) (k). Note that Caldesmon was also detected in the most basal epithelial cells (black arrowhead) in the gizzard (j). Scale bars = 50 micrometers.

Purified gastrokine-1 was used as antigen and injected in rabbit to produce a specific polyclonal antibody. This new antibody was able to detect gastrokine-1 by Western blot, as demonstrated in “Supplement S2” figure. The native form of gastrokine-1 was detected as a 20 kDa protein band, as shown by the correlation between the Coomassie blue stained gel pattern of the tryptic cleavage of gastrokine-1 presented on the left panel of “Supplement S2” figure and the Western blot detection of the same gel pattern after incubation with polyclonal antibody directed against purified gastrokine-1. But progressively with conversion of the 20 kDa protein band into the resistant 18 kDa gastrokine-1 cleaved product (as already shown in detail in “Figure 3”), there was only weak detection by this polyclonal antibody for this cleaved 18 kDa gastrokine-1 product. This led us assume that the major part of the epitopic determinant of this antibody was contained within the N-terminal part of gastrokine-1. In the following experiment, this antibody was used to detect gastrokine-1 in smooth muscle cross sections by using the immunofluorescence labelling technique. “Figure 4” shows the gastrokine-1 distribution during gastrointestinal development in chicken. In closer detail, gastrokine-1 was (“Figure 4”, parts a and c) present at the epithelium level (black arrowheads) in chick gizzard and duodenum, respectively. Alpha-SMA was not detected in a similar region, as shown by the serial sections in “Figure 4”, parts b and d, respectively. It is clear that areas corresponding to smooth muscle were stained with antibodies directed either against gastrokine-1 or α-SMA (red arrowheads). In addition, gastrokine-1 expression was observed in isolated islets (green arrow) present in the submucosa layer, but was not expressed in the enteric nerve cells (black arrows). Immunofluorescent detection patterns of gastrokine-1 (“Figure 4” part e) and α-SMA (“Figure 4” parts f) in E8 chick duodenum were similar in smooth muscle layers, as observed in the merged image (“Figure 4”, part g). To confirm these observations, immunostaining was compared in E16 chick gizzard and duodenum using antibody directed against either gastrokine-1 or another specific smooth muscle protein, i.e. caldesmon. Here too (“Figure 4”, parts h and k), gastrokine-1 was present in the gastric epithelium and also found to be abundant, rather granular, cytoplasmic globules (brown), at both the cell apex and bottom (black arrowheads). Similar areas were not detected as containing caldesmon. However, as shown with red arrowheads, the smooth muscle contained gastrokine-1 and caldesmon staining (“Figure 4”, parts i and j), and this was also observed in myofibroblasts in the lamina propria (green arrowhead) and in isolated islets (green arrow) present in the submucosa layer. Note that gastrokine and caldesmon were not expressed in enteric nerve cells (black arrows).

**Figure 5:**
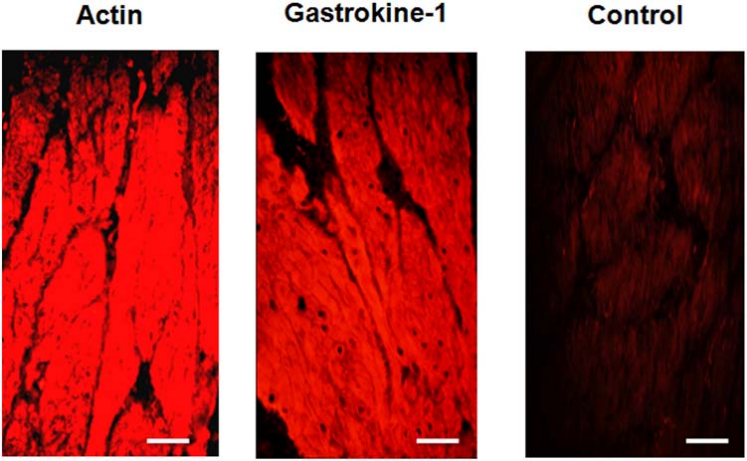
Codistribution of F-actin and gastrokine-1 in adult chicken gizzard smooth muscle Cryostat sections of adult chicken gizzard were stained using (left part) commercial anti-F-actin and (middle part) anti-gastrokine-1 that we produced as primary antibodies. Both proteins were similarly detected in the cytoplasm of the gizzard smooth muscle from adult chicken. An example of incubation with only the secondary fluorescent Cy3-linked fluorescent antibody when omitting either anti-actin or anti-gastrokine-1 primary antibody is shown on the right. Scale bars = 50 micrometers.

Then, and as expected from recent studies on genes induced during early developmental stages [21], gastrokine-1 was detected at E8 and E16 stages of the chick gizzard and duodenum. Similarly, the described gastrokine-1 localisation is in agreement with previous findings [1], [12] but, in addition, there was clear co-localisation with specific smooth proteins such as smooth actin-filaments and caldesmon in the gizzard, but also in chick duodenum. Using fresh sections of gizzard smooth muscle from adult chicken, labelling with smooth actin or with gastrokine-1 antibodies gave us, as presented in “Figure 5”, a similar distribution of both proteins in the cytoplasmic compartment of this muscle. A control is included in this figure, indicating that no fluorescent detection was achieved when each of the primary antibodies used (i.e. either directed against actin or gastrokine-1) was omitted from the first incubation step, but when the Cy3-linked specific fluorescent secondary antibody was still used. This also gave an indication of the background fluorescence level obtained after such incubation with only secondary antibody. This strongly suggests that gastrokine-1 could be a partner of F-actin in this tissue.

However, to confirm such co-distribution within smooth muscle, we used gastrokine-1 polyclonal antibody in other tissues and found, in agreement with recent related data on genes induced during early development stages [21], that gastrokine was completely absent in skeletal muscle. However, also in agreement with this work, we found that gastrokine-1 was weakly present in bovine heart and, more particularly, targeted to Hiss fibers (personal data, not shown). Although the presence of gastrokine in gastric epithelium is quite compatible with the actual focus of its relationship with only secretory products of mucous epithelia and its role in cytoprotection, it would certainly be less easy to explain the gastrokine presence in heart. This will be discussed later in the conclusion. Then in the following experiments we investigated the potential interaction between F-actin and gastrokine-1 in solution.

### Gastrokine-1 interact with F-actin

**Figure 6:**
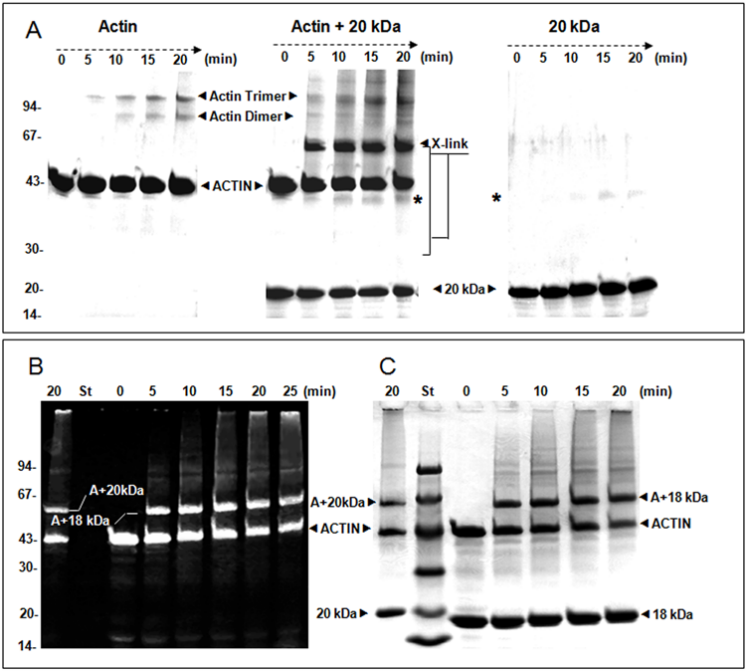
Kinetics of EDC cross-linking between F-actin and gastrokine-1 in solution compared to F-actin and gastrokine-1 alone. (A) Represent the Coomassie blue staining gel pattern of the covalent union induced by EDC treatment of F-actin alone (left part), F-actin and gastrokine-1 (middle part) and gastrokine-1 alone (right part). When only actin was submitted to EDC crosslinking conditions, actin-dimer and -trimer formation occurred. When only gastrokine-1 was mixed with EDC, GNK-1 dimer formation occurred, as shown by the label (*), and this entity was still present when the actin-gastrokine-1 mixture was treated with EDC. (B) A comparison of cross-linking time courses for native gastrokine-1 after 20 min of EDC treatment and that of trypsin split gastrokine-1 (stable 18 kDa fragment) when mixed with fluorescent F-actin is shown on the left. Note that only fluorescent bands appeared under UV light and indicated the presence of labelled actin within the fluorescent protein bands detected. Then, as shown on the right part, the same gel was stained was Coomassie blue and all protein bands were revealed. The 20 kDa protein band corresponding to gastrokine-1 was aligned with the corresponding molecular weight marker presented in lane St. The 18 kDa protein band corresponding to trypsin split gastrokine-1 was also viewed and new crosslinked protein bands were clearly identified according to their apparent molecular weights.

Taking advantage of the purified protein solution of the gastrokine-1 available in our laboratory, we tested the potential association with F-actin by the cross-linking approach using native or fluorescent actin (available in the lab). “Figure 6” shows a time-course analysis carbodiimide-dependent covalent association using EDC. As expected, the F-actin treated by EDC gave rise to actin dimer and trimmer (“Figure 6” panel A, left part). Gastrokine-1 alone led to the formation of a new protein band around 40 kDa corresponding to the dimer (“Figure 6” panel A, right part). Interestingly, we observed high accumulation of a new protein band that was suspected to be the actin-20 kDa product along with a relative decrease in F-actin dimer formation (“Figure 6” panel A, middle part). As shown in “Figure 6” (panel B), covalently associated proteins composing the protein band (expected to be an association of actin +20 kDa protein) contained fluorescent labelled actin (“Figure 6” panel B, left part). The same fluorescent gel was stained with Coomassie blue (“Figure 6” panel C, right part). The left side of the figure shows the result of the 20 min EDC treatment on the mixture of 20 kDa full length gastrokine-1 and F-actin. The covalent product contained fluorescent actin migrating at ~Mr of 63 kDa. On the right side of the standard protein markers, the complete time-course EDC treatment after Coomassie blue staining of the mixture of stable 18 kDa split gastrokine-1 and F-actin. The covalent new product also contained actin (according to the fluorescent labelling shown on the left side) and migrated as a protein band at around ∼61 kDa. Then we assumed that all new products initiated by EDC treatment could correspond to a covalent interaction between F-actin and native or split gastrokine-1. All of these covalent associated products were confirmed by Western blot using the polyclonal gastrokine-1 antibody. This is illustrated in “Supplement S3” figure to “Figure 6”. Three selected samples corresponding to native gastrokine-1 and the actin mixture submitted to the EDC crosslinking time course presented on the left part of the Western blot were compared after incubation with gastrokine-1 antibody and showed (on the right part of the figure) only protein bands containing gastrokine-1. Native gastrokine-1 was detected as a 20 kDa band, as expected and illustrated in “Supplement S2”. However, one major protein band was revealed with Mr 63 kDa. This new 63 kDa crosslinked protein band was revealed by specific fluorescence detection in actin and by Western blot using specific anti-gastrokine-1 antibody. This confirms that it resulted from the covalent union between actin and gastrokine-1. Another 40 kDa minor protein band was also present and assumed to be the gastrokine-1 dimer, as confirmed by Western blot revelation of the gastrokine-1 crosslink kinetics that will be presented later in part B of “Figure 8”. This could be preferentially induced by actin but also the result of oxidation of an older purified GNK1 preparation. In fact, as revealed later, the fresh GNK1 preparation alone did not induce dimer formation after EDC treatment.

### F-actin residues involved into gastrokine-1 association

**Figure 7:**
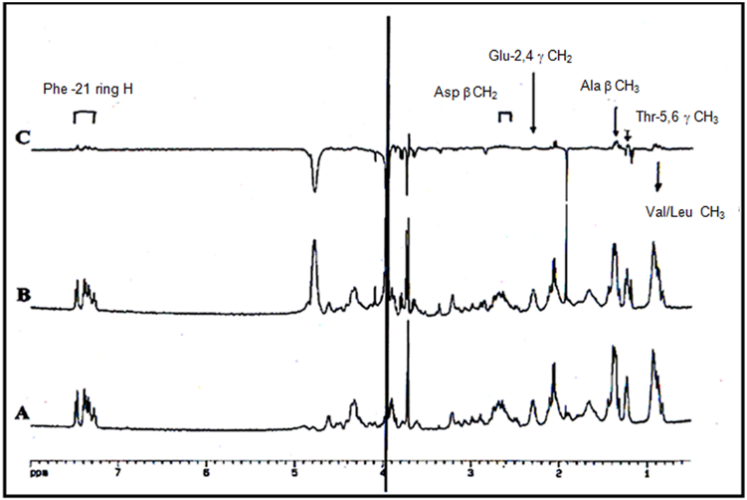
Interaction of the N-terminal region of actin with purified chicken GNK-1. Part (A): H-NMR spectrum of actin (200 mM) residues 1–28 containing 250 mM DTT, pH 6.9. Part (B): H-NMR spectrum after gastrokine-1 addition (25 mM) in similar conditions. Part (C): Difference spectrum (A–B) showing identification of the proton side chain belonging to specific actin residues that were perturbed upon gastrokine-1 addition and which are indicated in the corresponding area of the 1H-NMR spectrum. The identity of the protons involved, as well as the amino acid to which they belong, are given in a box over the corresponding peaks. The summary of this particular experiment is stated in the “Table 2” summarizes the results of this experiment along with other results obtained with different actin peptides.

To shed further light on the actin residues involved in the gastrokine-1 association, we used few actin peptides and tested interactions by the proton NMR technique. Each actin synthetic peptide was recorded in relation to its amino acid sequence (“Table 2”). Proton NMR spectroscopy was performed as detailed in a previous report [22]. Spectral perturbations resulting from complex formation between the synthetic actin peptides and gastrokine-1 were monitored by difference spectroscopy and by application of the two-pulse spin echo technique [22]. The most evident association was found between N-terminal peptide (residues 1 to 28) and gastrokine-1. “Figure 7” shows the respective records corresponding to the H-NMR spectra of N-terminal actin peptide alone (A), of the mixture of N-terminal peptide and gastrokine-1 (B) and the complex formation (C) which corresponds to the differential spectrum (A–B). Our data showed that the gastrokine-1/actin interface involves a specific proton belonging to well identified side chains of a few N-terminal actin residues. Although the proton mapping could not be precisely done for each residue, Glu-2 and -4 (E) as well as Thr- 5 and -6 or Phe-21 were clearly involved in the gastrokine-1 interaction (shown in bold in the N-terminal sequence of the 28 first actin amino acid residues). The other protons implicated in the gastrokine-1 association were in the side chain CH3 of Val/Leu and Ala or CH2 of Asp residues. According to these results, the underlined residues, mapped into the following actin sequence : D **E** D **ETT** ALVCDNGSGLVKAG**F** AGDDAPR, could correspond to the GNK1 actin binding site. This is in complete agreement with the EDC-induced covalent association between actin and gastrokine-1 which promotes the activation of actin acid residues for direct crosslink with gastrokine-1, as previously reported for myosin S1 and/or other actin binding proteins [23], [24], [25], [26].

**Table 2:** Synthetic peptide from skeletal actin used to map the gastrokine-1 interface using H-NMR spectroscopy.

Actin peptide identification	Actin Residues involved in GNK-1/ actin interaction
1 to 18	GLU 2 and 4; THR 5 and 6
1 to 28	GLU 2 and 4; THR 5 and 6; PHE 21
16 to 41	PHE 21
29 to 58	Not involved
58 to 84	Not involved
96 to 117	HIS 101
350 to 375	Not involved

All peptides were obtained as previously described [22]. Protons from clearly identified actin residues are listed as involved in contact between actin and gastrokine-1 and the most complete H-NMR differential spectrum is provided in “Figure S7”. When no differential spectrum was established, the corresponding peptide is indicated as not involved in actin/gastrokine-1 complex formation.

### Tropomyosin strengthening of gastrokine-1 and actin interaction

**Figure 8:**
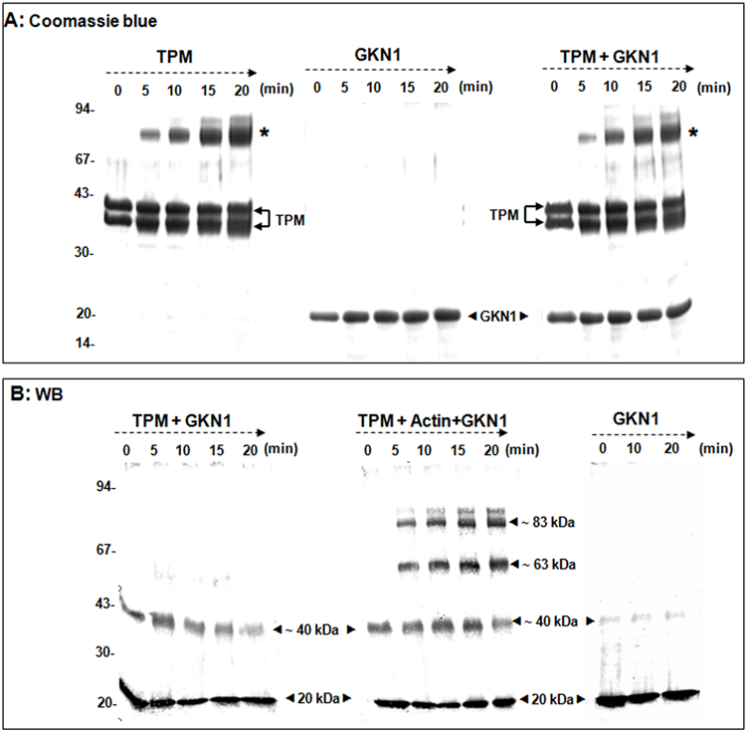
Importance of the tropomyosin presence during F-actin-gastrokine-1 association. EDC-induced protein stabilisation appeared as a new protein band as viewed after polyacrylamide gel electrophoresis. In part A, the gel is shown after Coomassie blue staining and at the time indicated, the kinetics of EDC cross-linked product formation is shown for tropomyosin alone (left panel), gastrokine-1 alone (middle panel) and a gastrokine-1 and tropomyosin mixture (right panel). Chicken gizzard tropomyosin appeared as a doublet band with a molecular weight of 37 and 40 kDa. No other new band than the 80 kDa product, highlighted with the label (*), was accumulated during the cross-linking experiment containing tropomyosin. Note that very fresh gastrokine-1 preparations did not accumulate any GNK-1 dimer upon EDC treatment. In part B, the gel was viewed by the Western blot technique using anti-gastrokine-1 that we produced. Only protein bands containing gastrokine-1 were viewed and, since all panels contained gastrokine-1, a protein band of 20 kDa appeared. The other protein bands had a molecular weight of around 40 kDa (gastrokine-1 dimer); 63 kDa (corresponding to the actin and gastrokine-1 association) and 83 kDa (covalent association between actin and gastrokine-1 dimers). The crosslinking conditions are indicated at the top of each panel and, in this panel, are tropomyosin and gastrokine-1 (left panel), ternary complex between gastrokine-1, actin and tropomyosin (middle panel) and gastrokine-1 alone (right panel).

With the aim of determining the potential involvement of gastrokine-1 in the regulation of the actin network in smooth muscle cells, we tested the effect of tropomyosin on the gastrokine-1/actin association. “Figure 8” (panel A) shows the Coomassie blue gel staining over the time course of EDC treatment of tropomyosin alone, chicken gastrokine-1 alone, and their mixture (ratio 1/1). Apart from a strong tropomyosin dimer with an Mr of about 80 kDa that appeared after 5 min of crosslinking, no other new protein band was clearly detected. After adding F-actin to this mixture, we observed that gastrokine-1, actin and tropomyosin could be associated within a ternary complex. To facilitate the interpretation of the EDC-induced covalent union, the cross-linking kinetics were electro-transferred and revealed by Western blotting analysis using polyclonal anti-gastrokine-1 (“Figure 8”, panel B). Three kinetics panels are presented and corresponded to the tropomyosin and gastrokine-1 mixture (left panel), the ternary complex obtained by the addition of F-actin (middle panel) and a control with gastrokine-1 alone. It is interesting to note that in the absence of actin or tropomyosin, gastrokine-1 was weakly converted into dimer form (∼40 kDa protein band) during EDC treatment. However, when mixed with tropomyosin or with the F-actin/tropomyosin mixture, gastrokine-1 dimer formation was induced before EDC addition. As expected, the presence of F-actin also led to the new 63 kDa covalently linked product previously described in our experiments. Moreover, we observed a new protein band with Mr ∼83 kDa. We logically assumed that this product corresponded to the covalent union between actin and the gastrokine-1 dimer. In fact, we noted a decrease in gastrokine-1 dimers during the treatment, whereas the 83 kDa product increased.

## Discussion

Many protein functions are currently deduced through comparative sequence homologies and theoretical prediction on the basis of cell compartment distributions. Here we described the gastrokine-1 protein structure and function using conventional biochemical approaches. The putative cellular function of gastrokine-1, a component of the protein family containing the BRICHOS domain, has been proposed [8]. Here we demonstrated the specific biochemical and cellular properties of chicken gastrokine-1. It was found that the N-terminal end of this protein was easily cleaved by proteolytic enzymes such trypsin and contained an enzyme resistant core of 18 kDa that was easily labelled by fluorescence directed against a so-called reactive cysteine residue. Gastrokine-1 was also able to form a dimer. It was previously described that this protein was expressed in normal stomach in the superficial/foveolar gastric epithelium [2], [3], [7]. Our findings showed that this protein was certainly present but was not restricted to this area of the stomach but was also present in smooth muscle cells. Down regulation of this protein in gastric carcinoma was postulated to be responsible for several diseases affecting the stomach [1], [27], but only the potential role of gastrokine-1 in mucosal protection was proposed. We provided evidence of a strong relationship of gastrokine-1 with actin and its potential role as a new locker and/or membrane anchoring protein for actin filamentous stress fibers involving the gastrokine-1 structure in the presence of two or possibly three predicted transmembrane helices.

In fact, cell-cell adhesion could involve the actin network via cadherin-catenin or integrin complexes within which gastrokine-1 could play a powerful role, and any weakening of these interactions could lead to a loss of cell-cell contact maintenance. The recent finding that gastrokine-1 and trefoil factors could have some interacting capacities is of interest, as recently reported [12]. Trefoil structures probably contribute to their resistance to protease degradation, but their role in cell-cell contacts is also evident [28]. Decreased expression of gastrokine-1 and the trefoil factor may thus favour the development of gastric cancer by weakening the actin assembly (stress fibers) and disrupting smooth cell-substratum contacts, or inducing loss of anchoring and thus anoikis, a form of apoptosis [29]. To determine whether other pathologies related to smooth muscle but also possibly to cardiac muscle may occur when gastrokine-1 is down-regulated, it is necessary to consider how the gastrokine-1/actin complex is involved in smooth muscle contractile performance–thus giving myosin a stronger (when GNK1 is present) but weaker (when GNK1 is absent) association with actin filaments and favoring modulation of stable actin-myosin complex formation. .Further research is needed to demonstrate this last point but already defects in smooth muscle performances must be investigated with regards to the potential involvement of gastrokine-1 in such related pathologies and the potential role of gastrokine-1 as a new potential actin-filament locker should also be considered in the light of the present data.

## Supporting Information

Supplement S1 Time course of limited trypsin cleavage of native purified gastrokine-1. *The gastrokine-1 protein was detected as a 20 kDa protein band before tryptic attack and progressively according to the time scale indicated at the bottom of the figure. Proteolytic cleavage led to a stable 18 kDa fragment*.
(6.42 MB TIF)

Supplement S2 Characterisation of the specificity of the anti-gastrokine-1 polyclonal antibody produced in this study. *The specificity of the polyclonal antibody obtained after injection of purified gastrokine-1 into rabbits was first tested against the native and freshly prepared protein. As expected, the purified sample of gastrokine-1 was revealed as a single protein band with an Mr of 20 kDa, but in the Western blot pattern of the tryptic cleavage of gastrokine-1 this antibody seemed to be less able to detect the 18 kDa stable cleaved product. This indicated that the major epitopic part of this antibody was related to the N-terminal extremity of gastrokine-1 which may be more accessible and also more susceptible to proteolytic cleavage.
(4.66 MB TIF)

Supplement S3 EDC crosslinked kinetics of gastrokine-1 and actin mixture revealed by Western blot. *Three examples of the EDC kinetics are presented after Coomassie blue staining on the left part of the figure. The specific anti-gastrokine-1 antibody that we produced was then used to investigate the new crosslinked products formed after EDC addition. Before the addition of EDC, only gastrokine-1 was detected as a 20 kDa protein band, as shown on the right part of the figure and then two protein bands were progressively revealed with Mr 40 kDa and 63 kDa. These new protein covalent entities were then identified as the gastrokine-1 dimer and the covalent union between gastrokine-1 and actin, respectively, and confirmed by the data presented in “Figure 6” using fluorescent actin. However, in others experiments, the formation of a 40 kDa protein bands was found as the induced tendency for the gastrokine-1 preparation to form GNK1 dimer when mixed with F-actin and/or tropomyosin preparation respectively, see western blot revealed in “Figure 8”, panel B)
(5.93 MB TIF)
