# Supplementary figures and images for: Biochemical Properties of Gastrokine-1 Purified from Chicken Gizzard Smooth Muscle
Source: PLoS One. 2008 Dec 5;3(12):e3854. doi: 10.1371/journal.pone.0003854 (PMC2588339; doi:10.1371/journal.pone.0003854)

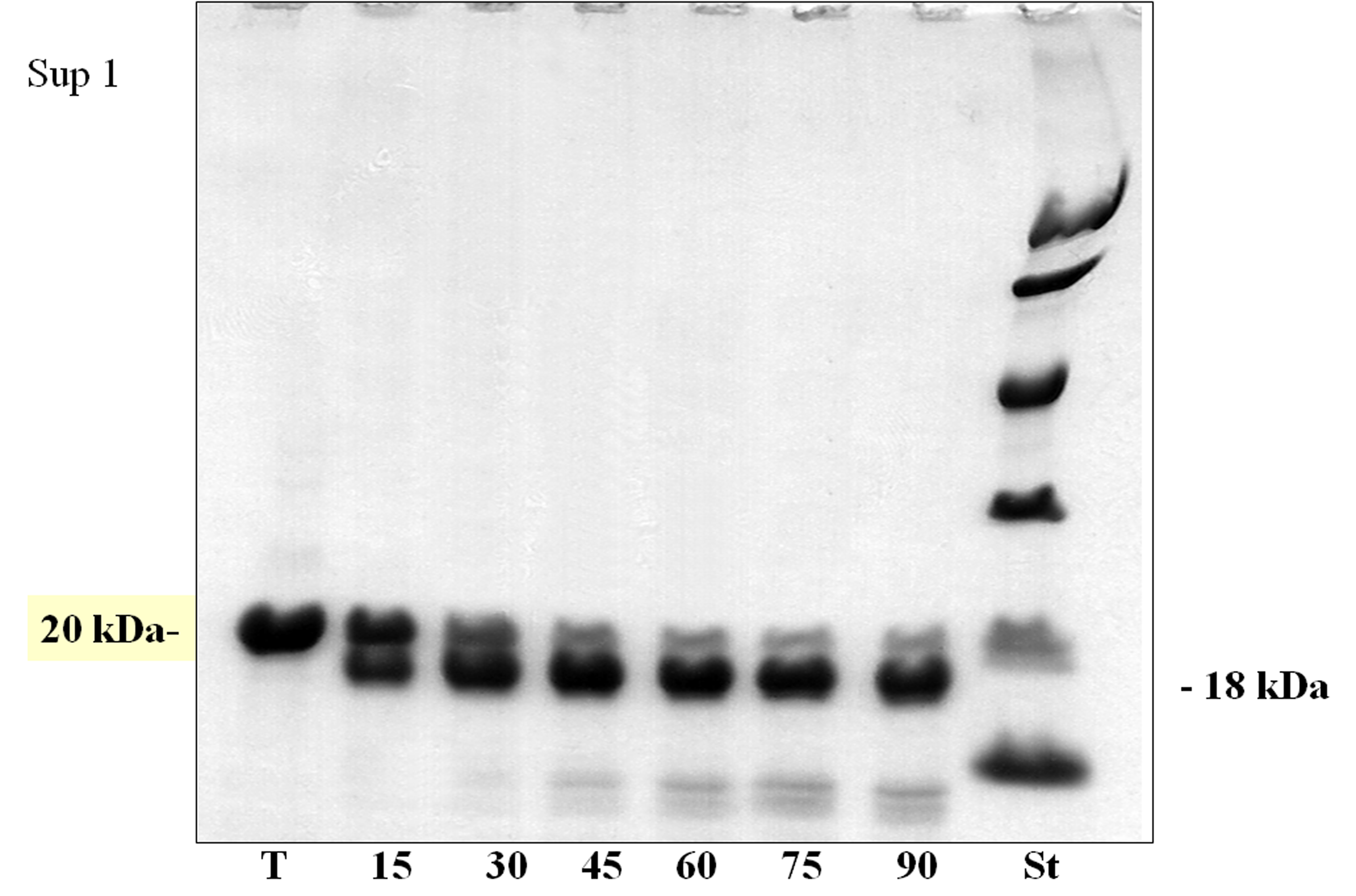

Supplement: Supplement S1 — Time course of limited trypsin cleavage of native purified gastrokine-1. *The gastrokine-1 protein was detected as a 20 kDa protein band before tryptic attack and progressively according to the time scale indicated at the bottom of the figure. Proteolytic cleavage led to a stable 18 kDa fragment*. (6.42 MB TIF) [file pone.0003854.s001.tif]

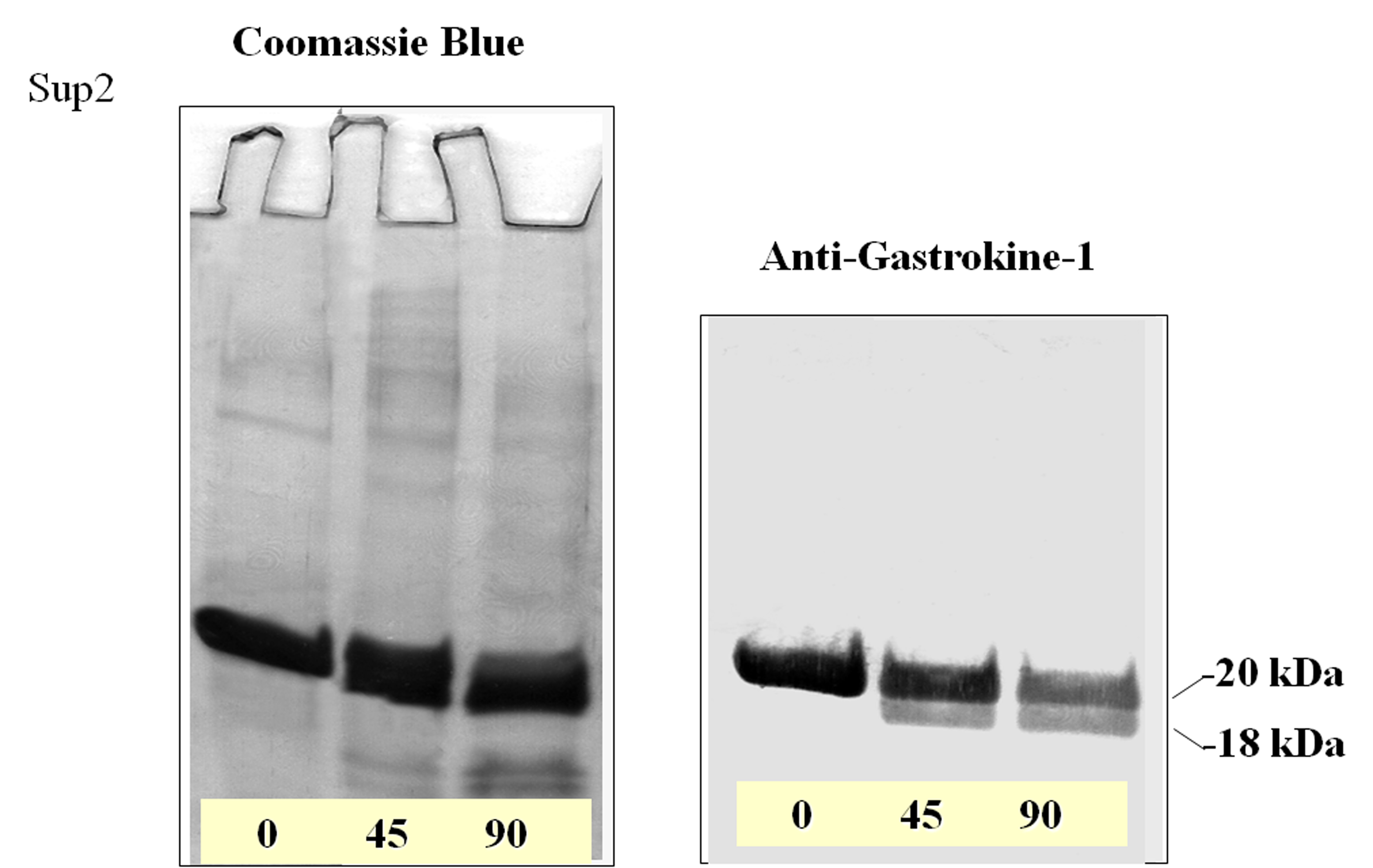

Supplement: Supplement S2 — Characterisation of the specificity of the anti-gastrokine-1 polyclonal antibody produced in this study. *The specificity of the polyclonal antibody obtained after injection of purified gastrokine-1 into rabbits was first tested against the native and freshly prepared protein. As expected, the purified sample of gastrokine-1 was revealed as a single protein band with an Mr of 20 kDa, but in the Western blot pattern of the tryptic cleavage of gastrokine-1 this antibody seemed to be less able to detect the 18 kDa stable cleaved product. This indicated that the major epitopic part of this antibody was related to the N-terminal extremity of gastrokine-1 which may be more accessible and also more susceptible to proteolytic cleavage. (4.66 MB TIF) [file pone.0003854.s002.tif]

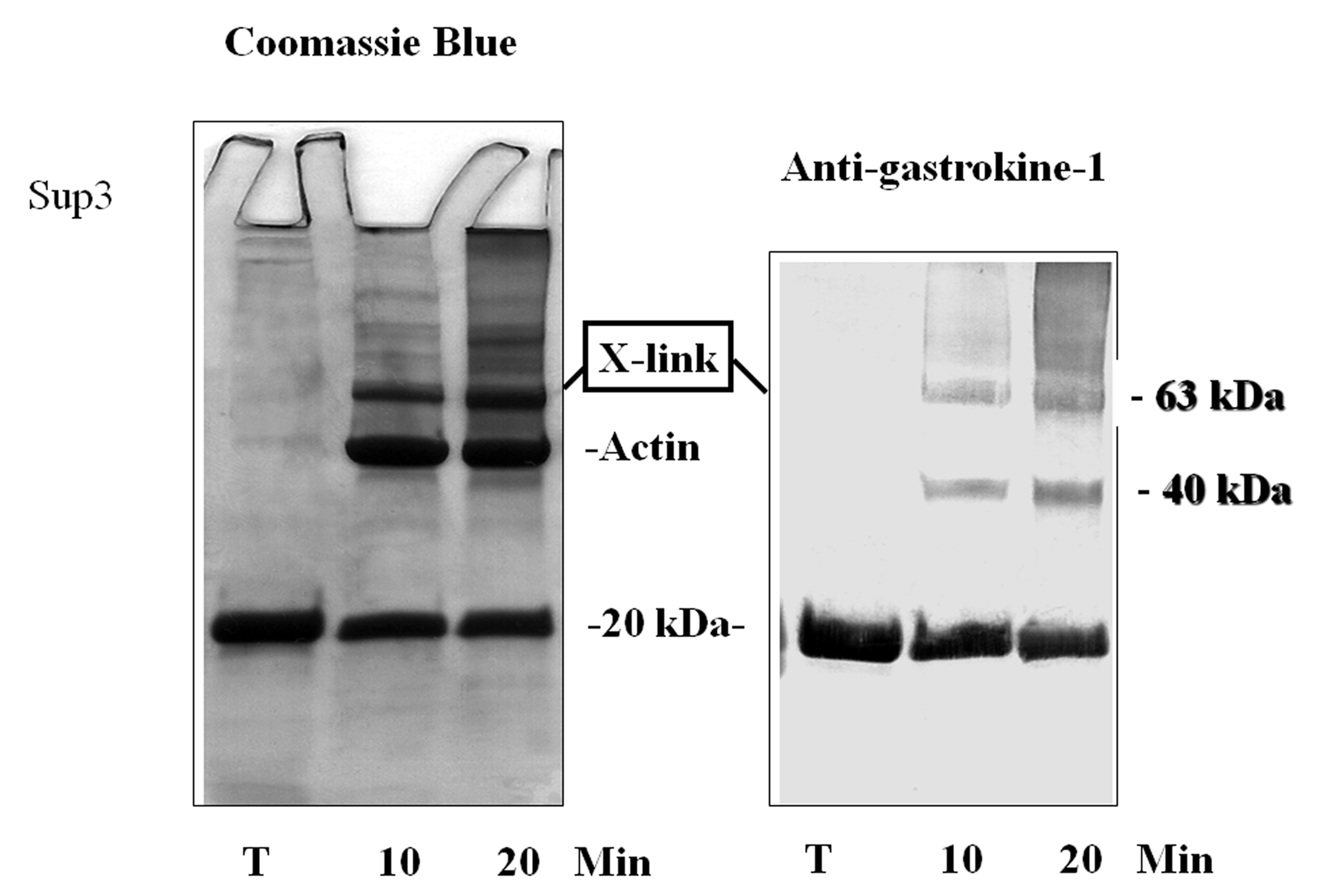

Supplement: Supplement S3 — EDC crosslinked kinetics of gastrokine-1 and actin mixture revealed by Western blot. *Three examples of the EDC kinetics are presented after Coomassie blue staining on the left part of the figure. The specific anti-gastrokine-1 antibody that we produced was then used to investigate the new crosslinked products formed after EDC addition. Before the addition of EDC, only gastrokine-1 was detected as a 20 kDa protein band, as shown on the right part of the figure and then two protein bands were progressively revealed with Mr 40 kDa and 63 kDa. These new protein covalent entities were then identified as the gastrokine-1 dimer and the covalent union between gastrokine-1 and actin, respectively, and confirmed by the data presented in “Figure 6” using fluorescent actin. However, in others experiments, the formation of a 40 kDa protein bands was found as the induced tendency for the gastrokine-1 preparation to form GNK1 dimer when mixed with F-actin and/or tropomyosin preparation respectively, see western blot revealed in “Figure 8”, panel B) (5.93 MB TIF) [file pone.0003854.s003.tif]
